# Fully Endoscopic Transforaminal Lumbar Discectomy for Upward Migration of Upper Lumbar Disc Herniation: Clinical and Radiological Outcomes and Technical Considerations

**DOI:** 10.3390/brainsci10060363

**Published:** 2020-06-10

**Authors:** Dong Hwa Heo, Dong Keun Lee, Dong Chan Lee, Hyeun Sung Kim, Choon Keun Park

**Affiliations:** 1 Department of Neurosurgery, Spine center, The Leon Wiltse Memorial Hospital, Suwon 16444, Korea; spinesurgery@naver.com (D.H.H.); vitamine-lee@hanmail.net (D.K.L.); allspine@gmail.com (C.K.P.); 2 Department of Neurosurgery, Gangnam Nanoori Hospital, Seoul 06048, Korea; neurospinekim@gmail.com

**Keywords:** lumbar, disc herniation, endoscopy

## Abstract

Microdiscectomy for the upward migration of upper lumbar herniated discs has a high risk of isthmus and facet injury. Fully endoscopic transforaminal discectomy can preserve normal bony structures during discectomy. The purpose of this study was to assess the clinical and radiological outcomes of fully endoscopic transforaminal discectomy for upward migrated upper lumbar herniated discs. All patients had upward migrated disc herniation from L1–L2 to L3–L4 levels and were treated using fully endoscopic transforaminal discectomy under local anesthesia. All enrolled patients were monitored for more than 12 months. Clinical outcomes were assessed using the Oswestry Disability Index (ODI) and visual analog scale (VAS) of pain. Surgery-related complications were analyzed. In addition, radiological outcomes were investigated using postoperative magnetic resonance imaging (MRI) and lumbar dynamic X-ray. Twenty-eight patients were enrolled in this study. ODI and VAS significantly decreased after endoscopic transforaminal discectomy. Migrated ruptured disc particles were completely removed and confirmed on postoperative MRI in 26 of the 28 patients. Even though small remnant disc particles were detected in two patients, symptoms improved after endoscopic transforaminal discectomy. Early recurrence of herniated disc occurred at the operated segment in one patient. There were no significant complications associated with fully endoscopic transforaminal discectomy. Three patients experienced a postoperative transient tingling sensation and numbness of the leg. Fully endoscopic transforaminal lumbar discectomy may be an effective and alternative treatment option for upward migrated disc herniation in the upper lumbar area. In addition, fully endoscopic transforaminal lumbar discectomy may prevent complications associated with general endotracheal anesthesia and injuries of the isthmus and the facet joint.

## 1. Introduction

The upper lumbar area is commonly defined as L1–L2, L2–L3, and L3–L4 [1,2]. The upper lumbar area is characterized by an anatomically narrow spinal canal and dense rootlets compared with the lower lumbar area. In addition, the lamina and the isthmus of the upper lumbar area are narrow compared with those in the lower lumbar area. Therefore, conventional open discectomy or microdiscectomy of upper lumbar disc herniation with upward migration is difficult and has a high risk of iatrogenic isthmic fracture, facet joint injury, and neurological damage including cauda equina injury [3]. Since only laminotomy with discectomy can produce procedural complications, fusion surgery could be an appropriate choice. Fusion procedures are considered in cases of upward ruptured disc or large disc herniation in the upper lumbar area due to a high risk of iatrogenic instability and neural injury [4].

Fully endoscopic transforaminal discectomy has been attempted in various types of lumbar degenerative diseases [5]. The fully endoscopic transforaminal approach was reported as a good treatment modality for upper lumbar disc herniation [6,7]. Lateral disc herniation and a young age were associated with an excellent outcome after fully endoscopic transforaminal discectomy for upper lumbar disc herniation [7]. The neural foramen in the upper lumbar area is wider and larger than that in the lower lumbar area. An upward migrated disc herniation can be removed using a transforaminal approach without removal of bony structure compared with downward migrated disc herniation. Therefore, the transforaminal endoscopic approach may be a good option for upward migration of disc herniation at the upper lumbar area. However, studies on upper lumbar disc herniation with upward migration are lacking.

Hence, the purpose of this study was to assess the clinical and radiological outcomes of the fully endoscopic transforaminal approach for the upward migration of upper lumbar foraminal disc herniation. In addition, we discuss technical considerations.

## 2. Materials and Methods

### 2.1. Patient Selection

This study defined the “upper” lumbar as L1–L2, L2–L3, and L3–L4. From September 2013 to December 2016, we performed 42 consecutive fully endoscopic transforaminal discectomies for upper lumbar disc herniation with upward migration. Surgical indications were as follows: (1) neurological symptoms and/or signs mostly in the legs and that were associated with the herniated disc at a single level and (2) intractable symptoms irrespective of non-surgical treatment, including epidural blocks for more than 6 weeks. Patients with concomitant central canal stenosis, foraminal stenosis, and instability were not considered for endoscopic transforaminal discectomy. Calcified disc herniations and downward migrating disc herniations were also excluded. We only enrolled patients monitored for more than 12 months. 

### 2.2. Analysis of Clinical Data

Clinical parameters such as demographic information, presenting symptoms, follow-up period, operation time, and complications associated with endoscopic procedures were retrospectively analyzed. Postoperative and last follow-up improvements in radicular leg pain and axial back pain were quantified using a visual analog scale (VAS) of the leg and the Oswestry Disability Index (ODI).

### 2.3. Classification of Upward Disc Migration and Analysis of Radiologic Parameters

**Figure 1:**
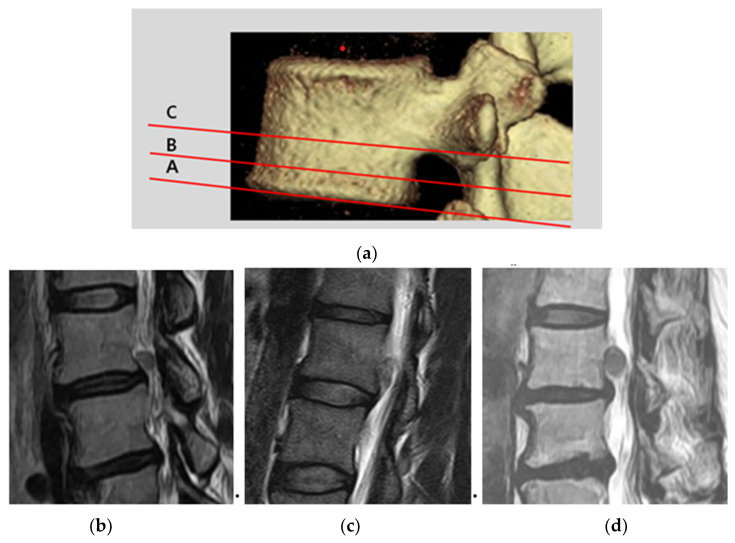
Classification of upward ruptured disc migration. (**a**) Type A, upward migration extent was less than half of the interval from the upper disc end plate line to the inferior pedicle margin; (**b**) type B, upward migration extent was more than half of the interval from the upper disc end plate line to the inferior pedicle margin; and (**c**) type C, upward migration extent was more than the lower margin of the pedicle (**d**).

Disc herniations, displaced away from the extrusion site, above the endplate level of the upper vertebral body were defined as upward migrated disc herniations [8]. We classified 3 types (A, B, and C) of migration zones (Figure 1a) using sagittal T2-weighted MRI. Type A represented herniations where the upward migration extent was less than half of the interval from the upper disc end plate line to the inferior pedicle margin (Figure 1b). Type B represented herniations where the upward migration extent was more than half of the interval from the upper disc end plate line to the inferior pedicle margin (Figure 1c). Type C represented herniations where the upward migration extent was more than the lower margin of the pedicle (Figure 1d, highly migrated disc herniation) [5,9].

We immediately performed postoperative MRI and postoperative X-rays. We investigated the degree of ruptured disc removal in the 3 types using immediate postoperative MR images. In addition, X-rays were taken at the final follow-up. Postoperative instability was investigated using flexion-extension dynamic X-ray films.

### 2.4. Surgical Technique

**Figure 2:**
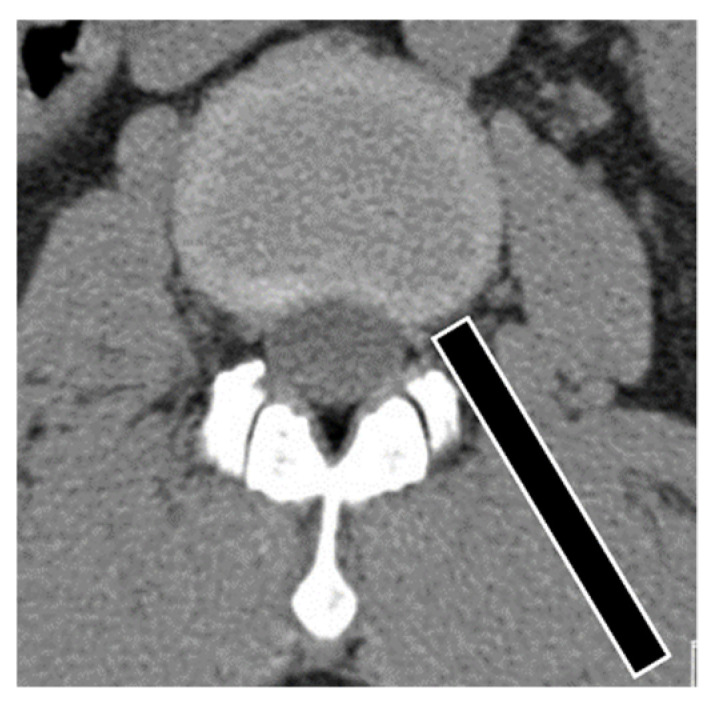
Angle of endoscopic trajectory for the upper lumbar area is steeper than that of the lower lumbar area for the prevention of neurological tissue injury.

The basic surgical technique for performing fully endoscopic transforaminal discectomy of the upper lumbar disc herniation was previously described [7]. For the transforaminal approach of the upper lumbar area, the skin entry point is typically approximately 7–10 cm from the midline, which is the more medial side than the lower lumbar, and caudal 0.5–1 cm from the midline disc space, which is the upper endplate margin of the inferior body. A steeper angle of needle insertion (from 30°–50°) is safer to prevent neural injury (Figure 2) in the anteroposterior projection and on the posterior vertebral line in the lateral projection. In our cases, after passing the exit root, epidural anesthesia was administered through the epidural catheter. Typically, a round-ended cannula was used for these types of herniations as it is easier to retract the exiting root and surrounding soft tissues. Next, the endoscope was placed in the epidural space outside the annulus. Through the endoscope, after removing the ligament flavum of the foramen, the surgeon identified a section of epidural fat, a landmark of the upper epidural space. Then, the tip of the working tube was lifted in the direction of the epidural space. The cannula was initially positioned at the lower part of the disc. After initial endoscopic exploration of the epidural space, the cannula was gradually shifted upward with a twisting motion until the exiting root was partially visible. Moving the cannula to the inferior margin of the pedicle was possible because the foramen is wider at the upper level. Movement of the cannula in the upper lumbar area was easier than in the lower lumbar area because the foramen is wider in the upper lumbar area. At this point, the ruptured fragment could be observed lying in the axilla, under the exiting root, partly covered by the superior foraminal ligament and ligamentum flavum. The release of soft tissue and ligament around the foramen exposed the ruptured disc particles. Herniated disc particles were removed using endoscopic pituitary forceps. After confirmation of the decompression of the exiting nerve root and pulsation of the dura, we finished the endoscopic procedures.

### 2.5. Statistical Analysis

This study design was a retrospective analysis of cases series. All parameters were analyzed using Mann–Whitney tests and Wilcoxon signed-rank tests. Multiple comparisons were analyzed using Kruskal–Wallis tests. Statistical differences were considered when *p* < 0.05. All data are expressed as the mean ± standard deviation. The analyses were performed using the R for Windows (version 3.6.3, R Foundation for Statistical Computing, Vienna, Austria).

## 3. Results

This study included 28 patients who were monitored for more than 12 months; 17 males and 11 females, ranging from 19 to 78 years (mean 51.1 ± 0.9 years). The mean follow-up period was 22.5 ± 6.9 months (Table 1).

**Figure 3:**
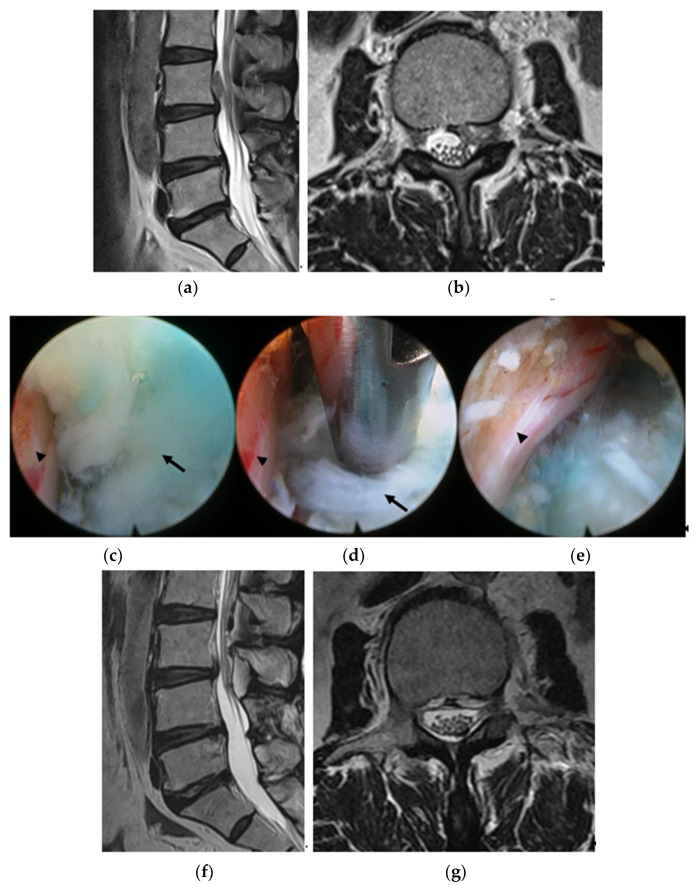
A 45-year-old man presenting with radiating pain in the left buttock and anterior thigh. Magnetic resonance imaging (MRI) revealed disc herniation with upward migration zone type C at L3–L4 (**a,b**). Ruptured disc particles were removed using the full endoscopic transforaminal approach (arrow: ruptured disc particles, arrow head: left side L3 nerve root, (**c,d**). After the full endoscopic transforaminal approach, the left side L3 nerve root was decompressed (**e**). A postoperative MRI showing that migrated ruptured disc particles were completely removed (**f,g**).

**Figure 4:**
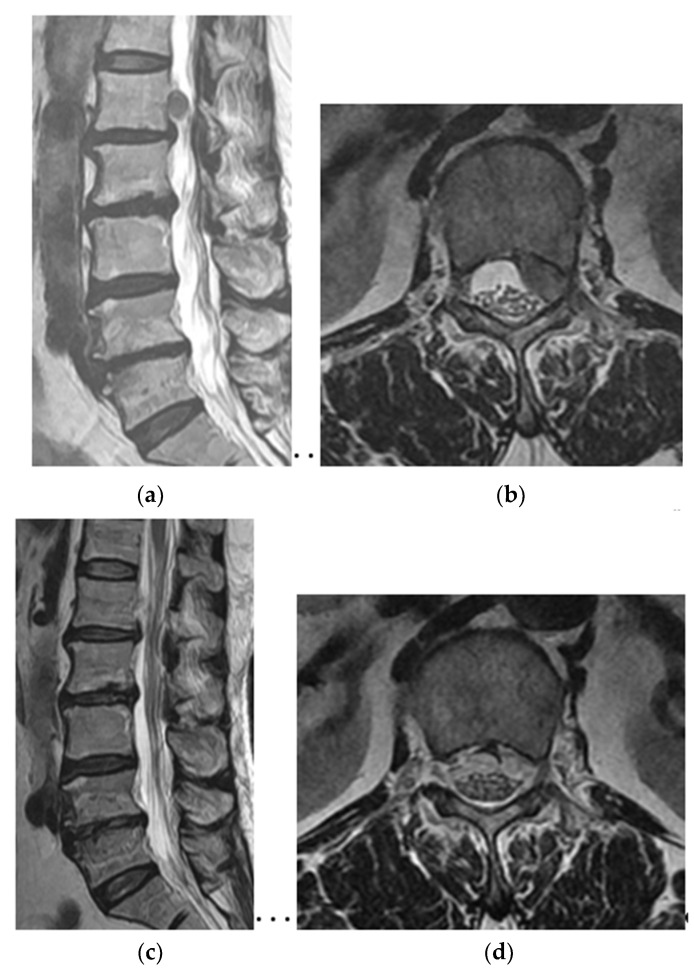
A 47-year-old man complained of radiating pain in the anterior thigh of the left leg. Preoperative MRI showed left-sided upward migrated disc herniation at L1–L2 (**a,b**). Ruptured disc fragments were removed using the fully endoscopic transforaminal approach (video S1). Although a small remnant disc particle was suspicious on postoperative MRI (**c,d**), radicular pain of left leg completely disappeared.

Distribution of upper lumbar disc herniation was as follows: L1–L2 (3 cases, 12%), L2–L3 disc (12 cases, 44%), and L3–L4 (13 cases, 44%). In addition, herniations with upward migration were localized at zone types A to C; 14 cases of zone type A, 8 cases of zone type B, and 6 cases of zone type C (Figure 3 and Figure 4, Table 1).

The mean operation time was 78 ± 6.6 minutes and was longer in zone type B (88.3 ± 48.2 min) and type C (90.0 ± 28.1 min) than in type A (67.7 ± 25.7 min). Although the mean operation time was longer in zone types B and C than type A, statistical difference was not observed between the zones (*p* > 0.05). Migrated ruptured disc particles were completely removed except in 2 zone type C cases. Although remnant disc material was detected in 2 zone type C patients, the particle size was very small, and symptoms were significantly improved after fully endoscopic transforaminal discectomy (Figure 4). One type A patient required reoperation due to recurrence on postoperative day 5.

In 23 of the 25 patients (92%), the ruptured disc fragments were completely removed based on postoperative MRI (Figure 3). In zone C, which was defined as upward migration of disc herniation above the lower margin of the pedicle, a small remnant disc particle was detected in two patients. However, the remaining tingling symptoms improved, even in those patients, a few days after endoscopic transforaminal discectomy.

VAS of the leg and ODI were significantly improved after endoscopic transforaminal discectomy. Preoperative VAS decreased from 8.4 ± 1.9 to 1.7 ± 1.5 on postoperative day 1 and to 2.2 ± 1.18 at the last follow-up (*p* < 0.05, Table 2). In addition, preoperative ODI decreased from 53.8 ± 15.5 to 21.1 ± 11.5 on postoperative day 1 and to 23.1 ± 9.2 at the final follow-up. One patient with a poor outcome presented early recurrence after a fully endoscopic transforaminal discectomy. A good outcome was obtained in the one patient with revision microdiscectomy after fully endoscopic transforaminal discectomy at the last follow-up.

There were no significant complications associated with the fully endoscopic transforaminal discectomy. One patient experienced transient numbness, and three noted a transient tingling sensation in the leg (Table 1). These symptoms were improved within 2 weeks of conservative treatment. Exiting root injuries such as dysesthesia, allodynia, or weakness associated with the permanent exiting root injury did not occur. Postoperative instability was not detected in any patient at the final follow-up.

## 4. Discussion

Endoscopic lumbar discectomy has been attempted as a minimally invasive alternative to conventional open discectomy for lumbar disc herniation [6,10,11,12]. However, application of this technique in the treatment of migrated disc herniation is limited [5]. Recently, many researchers have contributed in terms of technique and instrument development to resolve migrated disc herniation. Although techniques and instruments for endoscopic spine surgery have been developed, the fully endoscopic transforaminal approach of the highly migrated disc is still challenging and controversial. Fully endoscopic transforaminal discectomy for high-grade migrated disc herniation is technically difficult, and the clinical results may be less favorable [12,13].

Upper lumbar lesions, in particular, have narrow lamina, a wide facet joint, and a narrow isthmus. Posterior microdiscectomy can induce a facet fracture and isthmus injury during laminotomy [4]. Since the size of partial hemi-laminectomy for complete removal of herniated disc particles in cases of upward migrated disc herniation must be considered, isthmus injury is highly likely. Moreover, dura and root retraction during microdiscectomy may induce neural injury because the upper lumbar area contains dense rootlets compared with the lower lumbar area. Although fully endoscopic transforaminal lumbar discectomy required a longer period of learning curve and was technically unfamiliar compared to microdiscectomy, fully endoscopic transforaminal endoscopic may have advantages, especially in upward migration of upper lumbar disc herniation. First, fully endoscopic transforaminal discectomy does not require bone work and does not cause iatrogenic isthmic fractures in upper lumbar lesions. Second, the ventral approach in fully endoscopic transforaminal discectomy may minimize dura retraction. 

In cases of upward migrated disc in the upper lumbar area, because the size of the foramen in the upper lumbar area is larger and wider than in the lower lumbar area, a transforaminal endoscope can be smoothly moved to the upper portion of the foramen compared with the lower lumbar area. The anatomical features of the larger intervertebral foramen of the upper lumbar region may facilitate the removal of upward migrated disc herniation in the upper lumbar area. Moreover, a curved endoscopic hook and pituitary forceps can be used for removal of upward migrated disc materials.

Although endoscopic approaches have the advantage of being minimally invasive procedures without bone work, microdiscectomy, lumbar fusion surgery with wide laminectomy, or lumbar fusion surgery should be considered in cases with concomitant foraminal stenosis, segmental instability, and large central disc herniation for prevention of intraoperative neural injury. Reportedly, upper lumbar disc herniation requires fusion surgery [4]. The keyhole laminotomy approach or the transpedicular approach may be good alternative treatments for upward migrated lumbar disc herniation. If patients have severe disc space narrowing, foraminal stenosis, or a spinal deformity such as degenerative scoliosis, we suggest posterior endoscopic keyhole laminotomy or the transpedicular endoscopic approach as good surgical options.

Transforaminal endoscopic approaches have disadvantages associated with the procedures. Fully endoscopic transforaminal discectomy requires a long learning curve. Surgical anatomy and instruments for the transforaminal endoscopic approach are different from those of posterior lumbar microsurgery. Sufficient training for spinal endoscopic surgery is required for performing a complete transforaminal endoscopic approach. Another issue associated with the endoscopic approach is the possibility of incomplete surgery. The endoscopic approach we used was the out and in technique. The endoscope was first placed in the epidural space. The wide-sized upper lumbar foramen allowed easy upward movement of the endoscope in the epidural space and was sufficient to remove the migrated ruptured disc, including connected intradiscal material, using the endoscopic transforaminal discectomy technique. The wide-sized upper lumbar foramen may be a good predisposing factor for complete removal of the fragment in upward migrated disc cases. However, in most cases, the severely migrated herniations were multi-fragmented, and detecting such fragmentation on preoperative radiological studies is very difficult [12]. Intraoperative monitoring of C-arm fluoroscopy and patient response is important to prevent incomplete removal of the disc. In this study, upward migrated ruptured disc particles were completely removed except in two cases. Although two patients had partial remnant disc particles after fully endoscopic transforaminal discectomy, the particle size was very small, and the main symptoms were significantly improved in all patients.

Recurrence is an important concern. Traditionally, recurrence in the upper lumbar area is lower than in the lower lumbar area due to less mechanical loading and disc degeneration [14,15]. We experienced one case of recurrence after fully endoscopic transforaminal discectomy. Long-term follow-up and larger cohort studies are needed to investigate the recurrence rate associated with fully endoscopic transforaminal discectomy procedures.

Downward migrated ruptured disc is a very different situation compared with upward migration. Foraminoplasty or reaming procedures during fully endoscopic transforaminal discectomy were necessary for complete removal of downward migrated ruptured disc particles in the upper lumbar area. This situation is similar to downward migrated disc in the lower lumbar area. Therefore, only upward migrated ruptured disc cases in the upper lumbar area were analyzed in this study. The small number of cases and the short-term follow-up were the limitations in this study. To prove the efficacy of this procedure, studies with more patients and longer follow-up periods are necessary. Moreover, comparative studies with microdiscectomy should be performed.

## 5. Conclusions

Fully endoscopic transforaminal discectomy may be an effective and alternative treatment option for the upward migration of disc herniation in the upper lumbar area. From an anatomical perspective, fully endoscopic transforaminal discectomy is associated with a relatively wide foramen as well as a lower incidence of spinal stenosis. In addition, the results from this study indicate that upward migration of herniated disc in the upper lumbar area, treated with fully endoscopic transforaminal discectomy, may prevent complications associated with general endotracheal anesthesia and iatrogenic isthmic fractures as well as injury of the facet joint.

## Figures and Tables

**Table 1:** Demographic characteristics of 28 patients.

Group	Total
Patients (n)	28
Sex	
Male	17
Female	11
Age (years)	51.1 ± 0.9
Level	
L1–L2	3
L2–L3	12
L3–L4	13
Mean follow-up duration (months)	22.5 ± 6.9
Migration zone: Type	
A	14
B	8
C (highly migrated disc)	6
Complications	
Recurrent disc herniation	1
Transient numbness	1
Transient tingling sensation	3
Small remnant disc particles	2

**Table 2:** Clinical outcomes.

Parameter	Preoperative	Postoperative 1 day	Last follow-up
VAS of leg *	8.4 ± 1.9	1.7 ± 1.5	2.2 ± 1.8
ODI *	53.8 ± 15.5	21.1 ± 11.5	23.1 ± 9.2

* p < 0.05.

## Supplementary Materials

The following are available online at https://www.mdpi.com/2076-3425/10/6/363/s1, Video S1: An intraoperative endoscopic video showing percutaneous transforaminal endoscopic removal of ruptured disc particles at left L2–L3. A cannula was shifted to the upper pedicle area, and upward migrated disc particles were removed using endoscopic pituitary forceps under endoscopic view.
